# Elevated homocysteine levels, white matter abnormalities and cognitive impairment in patients with late-life depression

**DOI:** 10.3389/fnagi.2022.931560

**Published:** 2022-07-18

**Authors:** Huarong Zhou, Xiaomei Zhong, Ben Chen, Qiang Wang, Min Zhang, Naikeng Mai, Zhangying Wu, Xingxiao Huang, Xinru Chen, Qi Peng, Yuping Ning

**Affiliations:** ^1^Center for Geriatric Neuroscience, The Affiliated Brain Hospital of Guangzhou Medical University, Guangzhou, China; ^2^Department of Neurology, The Affiliated Brain Hospital of Guangzhou Medical University, Guangzhou, China; ^3^The First School of Clinical Medicine, Southern Medical University, Guangzhou, China; ^4^Guangdong Engineering Technology Research Center for Translational Medicine of Mental Disorders, Guangzhou, China

**Keywords:** late-life depression, cognitive impairment, elevated homocysteine levels, white matter abnormalities, interaction

## Abstract

**Background:**

Cognitive impairment in late−life depression (LLD) is considered to be caused by neurodegenerative changes. Elevated homocysteine (Hcy) levels may be linked to cognitive abnormalities associated with LLD. The important role of white matter (WM) damage in cognitive impairment and pathogenesis in patients with LLD has been widely reported. However, no research has explored the interrelationships of these features in patients with LLD.

**Objective:**

The goal of the study was to examine the interrelationship between Hcy levels, cognition, and variations in WM microstructure detected by diffusion tensor imaging (DTI) in patients with LLD.

**Methods:**

We recruited 89 healthy controls (HCs) and 113 patients with LLD; then, we measured the plasma Hcy levels of participants in both groups. All individuals performed a battery of neuropsychological tests to measure cognitive ability. Seventy-four patients with LLD and 68 HCs experienced a DTI magnetic resonance imaging (MRI) scan.

**Results:**

Patients with LLD showed significantly lower fractional anisotropy (FA) values in the bilateral inferior longitudinal fasciculus than those of healthy participants. Only in LLD patients was Hcy concentration inversely associated to FA values in the forceps minor. Finally, multiple regression analyses showed that an interaction between Hcy levels and FA values in the right cingulum of the cingulate cortex and right inferior longitudinal fasciculus were independent contributors to the executive function of patients with LLD.

**Conclusion:**

Our results highlight the complex interplay between elevated homocysteine levels and WM abnormalities in the pathophysiology of LLD-related cognitive impairment, consistent with the neurodegeneration hypothesis.

## Introduction

Late-life depression (LLD) is related to a number of neurocognitive deficits, including impaired global cognition, executive functioning, memory, attention, and visuospatial perception (Reinlieb et al., 2014; Zhang et al., 2021). It was stated that cognitive impairment in LLD is thought to be related to neurodegenerative changes (Invernizzi et al., 2021; Rhodes et al., 2021). Cognitive impairments have been identified as essential features of LLD, as they persist even after depressive symptoms have subsided and are strongly linked to poor functional and therapeutic results (Bhalla et al., 2006). Furthermore, according to the findings of two large-sample prospective cohort studies, older people with indications of depression had an estimated twofold greater risk of developing dementia (Katon et al., 2015; Kaup et al., 2016). Therefore, knowing the pathogenic cause of cognitive deficits in patients with LLD is critical for dementia prevention and for effective therapy.

According to an increasing number of studies, elevated homocysteine (Hcy) levels are associated with cognitive impairments and dementia (Setien-Suero et al., 2016; Smith and Refsum, 2016; Kim et al., 2019). Furthermore, a prospective cohort study meta-analysis identified that each 5 μmol/L rise in homocysteine level increased the comparative Alzheimer’s disease risk by 15 percent (Zhou and Chen, 2019). Since both LLD and elevated Hcy levels make individuals susceptible to cognitive deficits, comorbidity between these two disorders may lead to an increased prevalence and extent of cognitive deficits. A recent community-based cohort study discovered that there was a significant inverse correlation between Hcy levels and cognitive capability in elderly people with depressive symptoms (Ford et al., 2013).

Our recent report demonstrated that LLD patients had higher plasma Hcy levels and worse cognitive performance than those of controls. Furthermore, in LLD patients, plasma Hcy concentrations were observed to be negatively related to global cognition, visual space, attention, and executive function. More interestingly, when compared to those with LLD or high Hcy concentrations alone, elderly people with both high Hcy concentrations and LLD had more severe cognitive impairment (Zhou et al., 2020). However, the mechanisms behind the additive cognitive deficits caused by LLD and increased Hcy levels are not fully understood.

Recently, numerous investigations have indicated that abnormal white matter (WM) microstructure detected by means of diffusion tensor imaging (DTI) is significantly associated with LLD (Wen et al., 2014) and cognitive impairment (Li et al., 2013). Fractional anisotropy (FA) values are susceptible to changes in WM structure, such as axonal injury, myelin loss, edema, and cell death (Tae et al., 2018). Many studies have demonstrated that the FA value of LLD patients decreases in a large variety of fiber bundles and brain areas, indicating that the WM network of patients with LLD may be damaged, potentially leading to poor connectivity with gray matter (Wen et al., 2014; Harada et al., 2018). Moreover, WM fiber bundles maintain high-speed signal connections across various areas of the brain, and diminished WM integrity may contribute to cognitive impairment and clinical symptoms in patients with LLD (Shen et al., 2019; Rashidi-Ranjbar et al., 2020). Recently, numerous DTI researches have shown that the integrity of the WM was positively correlated with cognitive function in patients with LLD (Shimony et al., 2009; Yuan et al., 2010; Alves et al., 2012; Mettenburg et al., 2012). For instance, researchers have identified that abnormal WM microstructure in patients with LLD is connected with impairments in cognitive functions, including cognitive processing speed (Shimony et al., 2009), memory (Mettenburg et al., 2012), language (Alves et al., 2012), and executive function (Mettenburg et al., 2012). These findings imply that unconnected WM bundles or tracts may be involved in cognitive abnormalities in patients with LLD.

Interestingly, numerous investigations have shown an association between Hcy levels and WM abnormalities (Wright et al., 2005; Feng et al., 2013; Lee et al., 2017; Tan et al., 2018; Nam et al., 2019). For example, in people over 40 years old, Hcy level is a potential risk for WM injury (Wright et al., 2005). Another study showed that Alzheimer’s disease patients with high Hcy levels had lower FA values of WM bundles (Lee et al., 2017). Furthermore, a recent study illustrated that higher Hcy level was related to decreased WM volume and cognitive deterioration in healthy elderly individuals (Feng et al., 2013), implying that higher Hcy levels can affect cognitive performance and WM structure.

All of the above evidence indicates that either elevated Hcy levels or WM abnormalities may be correlated with cognitive impairment in LLD patients. However, to the best of our knowledge, the collaborative consequence of homocysteine and WM microstructure on cognitive performance in this specific population has not been investigated. Therefore, the current investigation was aimed to ascertain (1) whether FA values of WM fibers were altered in LLD patients and (2) the correlation between Hcy concentration and the FA value of WM bundles, as well as their combined effect on cognition in both patients with LLD and healthy controls.

## Materials and methods

### Participants

Patients with LLD were enrolled at the Affiliated Brain Hospital of Guangzhou Medical University. Advertisements in the public were used to attract healthy elderly people. All participants signed a written informed consent form after getting a comprehensive overview of the study. The Ethics Committee of the Affiliated Brain Hospital of Guangzhou Medical University approved the study.

The inclusion and exclusion criteria have been described in previous studies (Zhou et al., 2020). Briefly, the inclusion criteria were the following: (1) A DSM-IV Structured Medical Interview-based diagnosis of major depressive disorder and (2) age ≥ 60 years. The following were the exclusion criteria: (1) A history of other serious mental illnesses; (3) a family history of schizophrenia and/or bipolar disorder; (4) transcranial magnetic stimulation and any electroconvulsive treatment in the last 6 months; (5) neurological disorders, for instance, brain tumor and stroke; and (6) physical disorders, such as hypothyroidism and anemia, that may cause emotional problems. Healthy control (HC) individuals who were not depressed, who were at least 60 years old and who had normal cognition were included. They were all found to be free of psychiatric disease and had 17-item Hamilton Depression Rating Scale (HAMD-17) scores of less than 7 (Zimmerman et al., 2013). Other criteria for exclusion were identical to those used for individuals in the LLD group.

A total of one hundred thirteen patients with LLD and eighty-nine HCs were recruited. The demographic information of the patients has been described in our previous studies (Zhou et al., 2020). Briefly, no significant differences were found in sex, age, or educational years between participants in the HC and LLD groups.

### Neuropsychological evaluations

All of the participants completed a comprehensive battery of neuropsychological examinations as described in previous studies (Zhou et al., 2020). Briefly, the tests included the following six cognition domains: (1) Mini-Mental State Examination (MMSE) for global cognition; (2) Rey-Osterrieth Complex Figure (ROCF)-Delay Recall test and Auditory Verbal Learning Test (AVLT) for memory; (3) Trail Making Test (TMT)-B and Stroop Color and Word Test (SCWT)-C for executive function; (4) TMT-A and Symbol Digit Modalities Test (SDMT) for attention; (5) Verbal Fluency Test (VFT) and Boston Naming Test (BNT) for language ability; (6) Clock Drawing Test 4 (CDT4) and ROCF-Copy for visual space. The cognitive domain scores were determined by converting each test result to a standardized z score and taking the average of the total. Particularly, low scores implied high performance on exams that evaluate timing, such as SCWT-C, TMT-B, and TMT-A. As a result, before being transformed to the standard score, the scores were converted to the reciprocal (Shu et al., 2016).

### Plasma homocysteine concentration measurements

The Hcy measurements have been described in previous studies (Zhou et al., 2020). Briefly, fasting plasma Hcy concentrations were assessed using the enzyme cycling assay by automatic testers (AU5800 testers, Beckman Coulter, Brea, CA). All of the samples were analyzed by a research assistant who was blinded to the status of the subjects.

### Magnetic resonance imaging acquisition

Seventy-four patients with LLD and 68 HCs underwent MRI scans. MRI data were collected within 1 month of completing the neuropsychological evaluations. MRI data were obtained by means of a 3.0-Tesla Philips Achieva scanner (Philips, Best, Netherlands). Before DTI scanning, a T2weight image was taken to rule out major white matter lesions, tumors, and cerebral infarction. The participants were subjected to DTI with the following settings: Direction = 32, b0 = 1,000 s/mm^2^, echo time (TE) = 92 ms, repetition time (TR) = 10,015 ms, flip angle = 90°, field of view (FOV) = 256*256 mm^2^, imaging matrix = 128*128, voxel dimension of 2*2*2 mm^3^, and 75 contiguous slices.

### Data processing

All of the DTI images were obtained *via* the standard procedure PANDA software (a pipeline tool for analyzing brain diffusion images).^1^ PANDA is a MATLAB toolbox that incorporates FSL,^2^ Diffusion Toolkit^3^ and MRIcron^4^ (Cui et al., 2013). Each subject’s diffuse tensor data were skull-stripped and subjected to eddy current and head movement rectification. The directions of the diffusion gradients were adjusted. After that, each subject’s FA was calculated on a voxel-by-voxel basis. Individual FA pictures in native space were non-linearly registered to the FA template in Montreal Neurological Institute (MNI) space by means of the FNIRT command of FSL for normalization. After that, the mean of all aligned FA pictures was computed. We used an atlas-based segmentation strategy to examine diffusion changes in the major WM tracts. The FA maps of each subject were registered into the JHU WM Tractography Atlas (Hua et al., 2008). Twenty WM pathways were examined.

### Statistical analysis

The 20 WM tracts’ mean FA values of the were compared using analysis of variance (ANOVA), with age, education, and sex included as covariates. Partial correlation was used to discover the interrelationships between Hcy levels, cognition and FA values of the WM tracts, and control variables included age, sex, and years of education. Furthermore, stepwise multiple regression analysis was performed to explore the associations between Hcy levels, FA values, their interaction (Hcy × FA values) and cognitive functioning after adjusting for education, sex, age, and HAMD-17 scores. All data were examined by means of SPSS version 23.0 (IBM, Chicago, Illinois, United States). The significance levels were set at 0.05, and two-tailed significance values were used. False discovery rate (FDR) corrections described by Benjamini and Hochberg (1995) were used for multiple test corrections. After utilizing the Benjamini and Hochberg (1995) procedure, FDR-corrected *p*-values (i.e., *q*-values) lower than 0.05 were considered statistically significant.

## Results

### Group differences in fractional anisotropy values of atlas-based tracts

**FIGURE 1:**
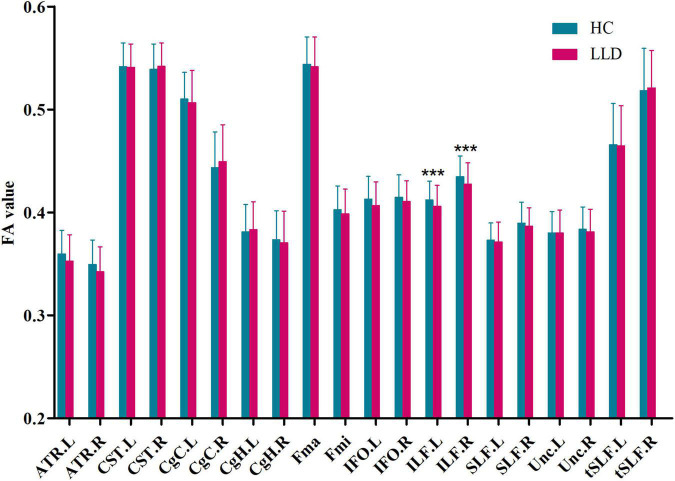
Group comparisons of the mean FA value of each tract in HCs and LLD patients. Each bar represents the mean ± SD. Analysis of covariance (ANCOVA) using age, sex, and education as covariates. *** indicates *q*< 0.05 after FDR correction. HC, healthy control; LLD, late-life depression; FA, fractional anisotropy; L, left, R, right; ATR, anterior thalamic radiation; CST, corticospinal tract; CgC, cingulum of the cingulate cortex; CgH, cingulum of the hippocampus; Fma, forceps major; Fmi, forceps minor; IFO, inferior fronto-occipital fasciculus; ILF, inferior longitudinal fasciculus; SLF, superior longitudinal fasciculus; Unc, uncinate fasciculus; tSLF, superior longitudinal fasciculus (temporal part).

Participants in the LLD group had significantly lower FA values in the left inferior longitudinal fasciculus (*F* = 6.17, *p* = 0.014, *q* = 0.014) and right inferior longitudinal fasciculus (*F* = 5.75, *p* = 0.018, *q* = 0.019) after adjusting for age, sex, and education than those of controls (Figure 1). However, there were no significant differences in the bilateral anterior thalamic radiation, bilateral corticospinal tract, bilateral cingulum of the cingulate cortex, bilateral cingulum of the hippocampus, forceps major, forceps minor, bilateral inferior fronto-occipital fasciculus, bilateral superior longitudinal fasciculus, bilateral uncinate fasciculus, or bilateral superior longitudinal fasciculus (temporal part) between participants in the HC and LLD groups (all *q* > 0.05).

### Relationships between cognitive deficits, homocysteine levels and fractional anisotropy values in healthy controls and patients with late−life depression

Hcy levels were significantly inversely correlated with FA values in the forceps major (Fma) only in patients with LLD after controlling for age, sex, and education (*r* = –0.31, *p* = 0.009, *q* = 0.009) (Table 1).

**TABLE 1:** Correlation of Hcy levels with FA values of WM tracts in patients with LLD.

	Hcy				Hcy		
	*r*	*p*	*q*		*r*	*p*	*q*
ATR.L	–0.13	0.281	0.468	IFO.L	–0.11	0.348	0.773
ATR.R	–0.13	0.301	0.602	IFO.R	–0.08	0.527	2.108
CST.L	–0.11	0.363	0.908	ILF.L	–0.14	0.241	0.321
CST.R	–0.14	0.263	0.376	ILF.R	–0.13	0.267	0.411
CgC.L	–0.18	0.139	0.164	SLF.L	–0.19	0.110	0.116
CgC.R	–0.17	0.173	0.216	SLF.R	–0.10	0.406	1.160
CgH.L	0.00	0.998	19.960	Unc.L	–0.06	0.602	3.010
CgH.R	–0.18	0.125	0.139	Unc.R	–0.05	0.674	4.493
Fma	–0.08	0.49	1.633	tSLF.L	–0.01	0.937	9.370
Fmi	–0.31	0.009	0.009	tSLF.R	–0.13	0.290	0.527

The false discovery rate (FDR) was adjusted using the Benjamini–Hochberg procedure, and q-values are reported; q-values less than 0.05 were considered significant. Adjusted for age, sex, and years of education. Hcy, homocysteine; L, left; R, right; ATR, anterior thalamic radiation; CST, corticospinal tract; CgC, cingulum of the cingulate cortex; CgH, cingulum of the hippocampus; Fma, forceps major; Fmi, forceps minor; IFO, inferior fronto-occipital fasciculus; ILF, inferior longitudinal fasciculus; SLF, superior longitudinal fasciculus; Unc, uncinate fasciculus; tSLF, superior longitudinal fasciculus (temporal part).

**FIGURE 2:**
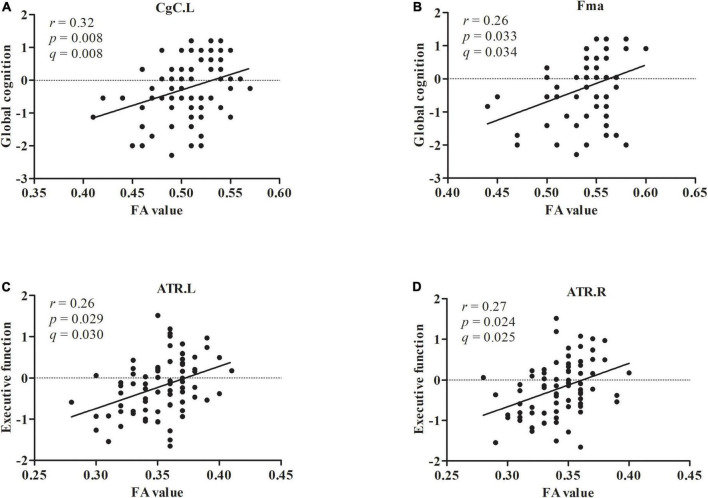
The FA value in CgC.L and Fma displayed positive associations with global cognition **(A,B)**. The FA value in ATR.L and ATR.R displayed positive associations with executive function **(C,D)**.

As shown in Figure 2A, the left cingulum of the cingulate cortex (*r* = 0.32, *p* = 0.008, *q* = 0.008) displayed positive associations with global cognition in patients with LLD after adjusting for age, sex, education, and HAMD-17 scores. As shown in Figure 2B, the forceps major (*r* = 0.26, *p* = 0.033, *q* = 0.034) displayed positive associations with global cognition in patients with LLD after controlling for age, sex, education, and HAMD-17 scores. The left anterior thalamic radiation (*r* = 0.26, *p* = 0.029, *q* = 0.030) displayed positive associations with executive function in patients with LLD after adjusting for covariates (Figure 2C). The right anterior thalamic radiation (*r* = 0.27, *p* = 0.024, *q* = 0.025) displayed positive associations with executive function in patients with LLD after adjusting for covariates (Figure 2D). There was no correlation between FA values and other cognitive domains.

Furthermore, no correlation between FA values and Hcy levels or between FA values and cognitive performance was found in HCs (all *q* > 0.05).

### Interaction of homocysteine level and fractional anisotropy values with cognition in patients with late−life depression and healthy controls

Multiple regression analysis displayed that the Hcy × FA interactions in the right cingulum of the cingulate cortex (beta = –0.24, *t* = –2.06, *p* = 0.043, *q* = 0.044) and right inferior longitudinal fasciculus (beta = -0.24, *t* = -2.12, *p* = 0.038, *q* = 0.038) were independent predictors for executive function only in patients with LLD. No association between the Hcy level × FA interaction in any of the 20 WM fiber tracts and other cognitive domains was found in patients with LLD (all *q* > 0.05).

Furthermore, no association between the Hcy level × FA interaction in any of the 20 WM fiber tracts and cognition was found in HCs (all *q* > 0.05).

## Discussion

The findings from the current study showed (1) significantly lower FA values in the bilateral inferior longitudinal fasciculus in patients with LLD than those in HCs; (2) disruption of WM structure was linked to elevated Hcy concentrations and cognitive impairments in global cognition and executive function in LLD patients; and (3) the interaction between elevated Hcy concentrations and structural destruction of WM may affect executive deficits in patients with LLD.

### Elevated homocysteine concentrations and white matter abnormalities in patients with late−life depression

Our previous report demonstrated that LLD patients had higher plasma Hcy levels and worse cognitive performance than those of healthy controls. Furthermore, higher plasma homocysteine levels were associated with poorer cognition in patients with LLD (Zhou et al., 2020). In the current study, we discovered lower FA values in the bilateral inferior longitudinal fasciculus in LLD patients, which is consistent with the majority of prior findings on FA values in patients with LLD (Alves et al., 2012; Mettenburg et al., 2012; Guo et al., 2014; Li et al., 2014, 2020; Wen et al., 2014; Emsell et al., 2017), confirming the LLD disconnection theory. Furthermore, we recognized a reverse relationship between plasma Hcy concentrations and FA values in the forceps minor in patients with LLD, suggesting that Hcy level is related to the WM structure. Because this is the first study to explore the increased homocysteine level and WM abnormalities of patients with LLD, any causal inferences are speculative.

Previous reports revealed that high Hcy concentrations inhibit mitochondrial activity in brain cells (Zhang et al., 2020), which might explain the link between hyperhomocysteinemia and cognitive impairment. In the developing brain, homocysteine induces cell cycle disruption and reactive gliosis (Cecchini et al., 2019). Data from animal studies demonstrate that even a slight increase in serum homocysteine levels upregulates matrix metalloproteinase-9 expression levels and disrupts blood–brain barrier integrity (Chu et al., 2021). In addition, endothelial dysfunction may result from an elevation in Hcy levels by enhancing oxidation and endothelial cell activation (Faverzani et al., 2017), increasing levels of adhesion molecules and proinflammatory cytokines (Barroso et al., 2016), and decreasing vascular wall integrity (Jakubowski, 2001). Endothelial cell damage causes a disruption in the tissue milieu, which leads to subsequent myelin injury and neurodegeneration. Moreover, a recent study demonstrated that increased Hcy levels were related to the imaging burden of cerebral small vessel disease (Cao et al., 2021). Taken together, animal and human research has suggested that high Hcy levels may affect adult neurogenesis as well as neuronal structure and function.

### Interaction of elevated homocysteine levels and white matter abnormalities in cognitive deficits in patients with late−life depression

Our findings revealed a negative relationship between plasma Hcy levels and FA values in the forceps minor in patients with LLD, which reflects the interaction between elevated Hcy levels and abnormal WM structure as the pathogenic mechanism of LLD. Moreover, we found that the Hcy × FA interaction in the right cingulum of the cingulate cortex and right inferior longitudinal fasciculus contributed to executive dysfunction in patients with LLD. Executive deterioration is common in patients with LLD and predicts a worse response to antidepressant therapy as well as a greater recurrence risk (Alexopoulos et al., 2005). Growing evidence has shown that high levels of homocysteine and WM degeneration are associated with cerebrovascular disease (Kynast et al., 2018; Cao et al., 2021). Therefore, elevated Hcy levels and WM abnormalities support vascularity impairment in patients with LLD. Although these correlations do not provide proof of causality in pathophysiology, it is speculated that high Hcy levels may disrupt WM microstructural connections and induce cognitive impairment. Additionally, our findings showed that there was a positive association between global cognition and FA values in the left cingulum of the cingulate cortex or the forceps major, as well as between executive function and FA values in the bilateral anterior thalamic radiation in patients with LLD. Several investigations have described that neurocognitive impairment is related to WM abnormalities in LLD (Charlton et al., 2014; Respino et al., 2019; Wang et al., 2020). These findings support the hypothesis that damaged WM connections are related to cognitive abnormalities in patients with LLD.

## Limitations

This research investigation had several limitations that must be noted. First, this was a case-control study. Thus, the causal relationship between elevated Hcy levels, WM injury and cognitive deficits in LLD patients is still uncertain. Therefore, in the future, a longitudinal investigation with a larger sample size will be required to obtain more persuasive and accurate results. Second, our research only investigated changes in WM structure; however, whether the change in WM structure is related to a change in function must be further explored. Third, the study did not assess diet, physical activity or lifestyle. All of these factors may influence Hcy levels and WM, but they were not controlled for in the present analysis.

## Conclusion

In conclusion, both increased Hcy levels and the disturbance of WM structure may be involved in the cognitive impairment observed in patients with LLD. Only in the patient group was a negative relation between plasma Hcy levels and WM disconnectivity identified, implying pathogenic processes underlying the interaction between increased Hcy levels and WM disconnection. Furthermore, because our current investigation used a case-control methodology, a larger longitudinal sample is needed to identify the causal relationship among aberrant Hcy metabolism, WM disconnection, and cognitive deficits in patients with LLD.

## Data availability statement

The raw data supporting the conclusions of this article will be made available by the authors, without undue reservation.

## Ethics statement

The studies involving human participants were reviewed and approved by the Ethics Committee of Affiliated Brain Hospital of Guangzhou Medical University. The patients/participants provided their written informed consent to participate in this study.

## Author contributions

YN conceived and designed the study. HZ and XZ performed testing and data collection and drafted the manuscript. BC, QW, MZ, NM, ZW, XH, XC, and QP performed the data analysis and interpretation. All authors contributed to the article and approved the submitted version.

## Conflict of interest

The authors declare that the research was conducted in the absence of any commercial or financial relationships that could be construed as a potential conflict of interest.

## Publisher’s note

All claims expressed in this article are solely those of the authors and do not necessarily represent those of their affiliated organizations, or those of the publisher, the editors and the reviewers. Any product that may be evaluated in this article, or claim that may be made by its manufacturer, is not guaranteed or endorsed by the publisher.

## Footnotes

1
http://www.nitrc.org/projects/panda/

2
https://fsl.fmrib.ox.ac.uk/fsl/fslwiki

3
http://www.trackvis.org/dtk

4
https://www.nitrc.org/projects/mricron
